# Cancer hotspot mutations rewire ERK2 specificity by selective exclusion of docking interactions

**DOI:** 10.1016/j.jbc.2025.108348

**Published:** 2025-02-25

**Authors:** Jaylissa Torres Robles, Amy L. Stiegler, Titus J. Boggon, Benjamin E. Turk

**Affiliations:** 1 Department of Chemistry, Yale University, New Haven, Connecticut, USA; 2 Department of Pharmacology, Yale School of Medicine, New Haven, Connecticut, USA; 3 Department of Molecular Biophysics and Biochemistry, Yale University, New Haven, Connecticut, USA

**Keywords:** protein kinases, mitogen-activated protein kinases, short linear motifs, substrate specificity, cell signaling

## Abstract

The protein kinase ERK2 is recurrently mutated in human squamous cell carcinomas and other tumors. ERK2 mutations cluster in an essential docking recruitment site that interacts with short linear motifs found within intrinsically disordered regions of ERK substrates and regulators. Cancer-associated mutations do not disrupt ERK2 docking interactions altogether but selectively inhibit some interactions while sparing others. However, the full scope of disrupted or maintained interactions remains unknown, limiting our understanding of how these mutations contribute to cancer. We recently defined the docking interactome of wild-type ERK2 by screening a yeast two-hybrid library of proteomic short linear motifs. Here, we apply this approach to the two most recurrent cancer-associated mutants. We find that most sequences binding to WT ERK2 also interact with both mutant forms. Analysis of differentially interacting sequences revealed that ERK2 mutants selectively lose the ability to bind sequences conforming to a specific motif. We solved the co-crystal structure of ERK2 in complex with a peptide fragment of ISG20, a screening hit that binds exclusively to the WT kinase. This structure demonstrated the mechanism by which cancer hotspot mutations at Glu81, Arg135, Asp321, and Glu322 selectively impact peptide binding. Finally, we found that cancer-associated ERK2 mutations had decreased activity in phosphorylating GEF-H1/ARHGEF2, a known ERK substrate harboring a WT-selective docking motif. Collectively, our studies provide a structural rationale for how a broad set of interactions are disrupted by ERK2 hotspot mutations, suggesting mechanisms for pathway rewiring in cancers harboring these mutations.

The RAS-RAF-MEK-ERK signaling cascade is activated downstream of growth factor receptors and is integral to regulating cell growth, proliferation, and survival (1). Oncogenic mutations in the RAF and RAS gene families found in approximately 30% of human cancers constitutively activate the pathway, and inhibitors targeting each of the core components of the cascade are in current use or in clinical trials as anti-cancer drugs (2, 3). While less frequent than mutations of upstream components of the pathway, recurrent mutations in the *MAPK1* gene encoding ERK2 are found in ∼8% of cervical squamous cell carcinomas, ∼1% of head and neck squamous cell carcinomas (HNSCC), and occasionally other tumor types (4, 5, 6, 7). *MAPK1* mutations at overlapping sites are also associated with a rare neurodevelopmental RASopathy disorder (Noonan Syndrome 13) (8). In HNSCC the most recurrent mutation, ERK2^E322K^, has been associated with exceptional response to the epidermal growth factor receptor (EGFR) inhibitor erlotinib, suggesting ERK mutation may predict susceptibility to similar agents in current clinical use (9, 10). How ERK2 mutation sensitizes cells to EGFR inhibitors is unclear, partly because the full impact of these presumptively gain-of-function (GOF) mutations is unknown (11).

Unlike cancer-associated mutations in other kinases, recurrent ERK2 mutants are not intrinsically hyperactive. Though distributed throughout the primary sequence, the most recurrent mutations (E81K, R135K, D321N, and E322K) map to a three-dimensional hotspot at the so-called common docking (CD) site within the ERK2 catalytic domain (Fig. 1, *A* and *B*). This site is located at one end of the docking recruitment site (DRS), a shallow groove opposite the catalytic cleft that serves as a hub for interactions integral to ERK signaling (Fig. 1*B*) (12). The DRS binds to short linear sequence motifs termed docking sites (or D-sites), often found in intrinsically disordered regions of interacting proteins (13). The DRS comprises a negatively charged CD site and four hydrophobic pockets (Φ_A_ ,Φ_B_, Φ_L_, Φ_U_). Complementary to the DRS, validated docking sites generally conform to a consensus sequence consisting of a basic (β) patch linked to a hydrophobic (ϕ) motif by a variable linker (β_2-3_-x_1-6_-ϕ-x-ϕ). A number of docking motif classes have been previously described based on their bound conformations and selectivity toward ERK and other mitogen-activated protein kinases (MAPKs) (14, 15, 16, 17, 18). Functionally important docking sequences are found among ERK activators (MAP2K1/2, collectively called MEK), substrates (RSK1, ELK1), and inactivating phosphatases (dual specificity phosphatases [DUSPs], and protein tyrosine phosphatases [PTPs]). Accordingly, the DRS is considered essential to ERK signaling.

**Figure 1:**
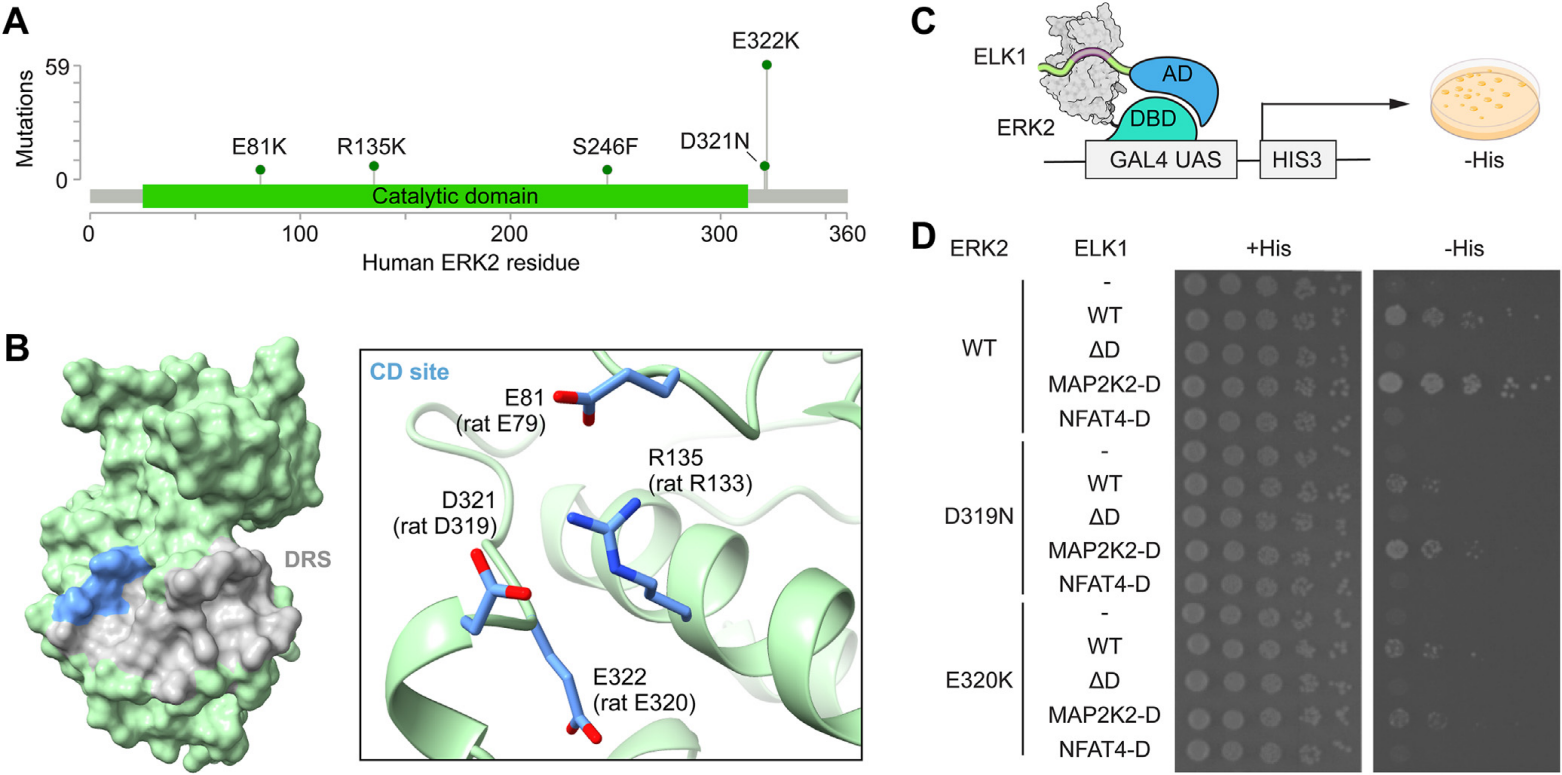
**Detecting interactions with ERK2 hotspot mutations by Y2H growth assay.***A*, lollipop diagram generated using the cBioPortal (6) MutationMapper tool shows *MAPK1* missense mutations observed in at least 5 tumors as reported in the COSMIC database (53). *B*, ERK2 structure (PDB accession code: 2ERK) with the DRS shaded and CD site indicated in blue. The inset shows sites of cancer-associated hotspot mutations in the CD site (blue sticks). *C*, scheme of the docking interaction-dependent ERK2-ELK1 Y2H system. *D*, Y2H spotting assay on solid media. Spots show growth of serially diluted cultures of strains co-expressing indicated variants of ERK2 and ELK1 under selective (−His + 50 μM 3-AT) and non-selective (+His) conditions.

While mutation of a key interaction hub might be expected to be disruptive, multiple lines of evidence suggest that ERK2 docking groove mutations are instead GOF. ERK2^D321N^ for example recapitulates the *sevenmaker* mutation, a GOF allele of the *Drosophila melanogaster* ERK ortholog identified in a forward genetic screen for modulators of growth factor signaling (19). Furthermore, in comparison to the WT allele, ERK^E322K^ had an increased ability to transform fibroblasts, and mouse ERK2^D319N^ (equivalent to human D321N) had enhanced capacity to induce epithelial-to-mesenchymal transition, cell invasion, and transcription factor activation in breast epithelial cells (20, 21). In addition, a saturation mutagenesis screen in melanoma cells for GOF ERK2 alleles returned all cancer hotspot mutations, and docking groove mutations can promote resistance to BRAF or MEK inhibitors (5, 22). The prevailing model for why these mutants are GOF is that their impact on docking interactions is selective: while some binding to MEK is maintained, there is effectively complete disruption of docking to both DUSP and PTP-type MAPK phosphatases including the key negative regulator MKP3 (DUSP6) (5, 21, 23, 24, 25, 26, 27). However, it is also the case that hotspot mutations cause selective loss of interactions with substrates. For example, ERK2 mutants fail to interact with RSK1, a major ERK substrate thought to have critical roles in cell growth and survival, yet maintain phosphorylation of ELK1, MBP, and c-Jun (5, 24, 28, 29). Decreased phosphorylation of some substrates may explain why the ERK2^D321N^ mutation can impair function in some cases, for example, in driving resistance to KRAS inhibition in pancreatic cancer cells (30). Collectively, these prior observations suggest that cancer-associated ERK2 mutations rewire signaling output, resulting in partial GOF that can support tumor growth in some contexts. How these mutations change ERK2 docking site specificity is fundamental to understanding their contribution to promoting cancer and drug susceptibility, but there is currently no way to predict which interactions will be broken and which will be spared.

To determine the scope of interactions maintained or disrupted by cancer-associated ERK2 mutants, in this study, we screened a library of thousands of human protein fragments to identify those binding differentially to WT and mutant ERK2. We found that most sequences interacting with WT ERK2 are maintained upon ERK2 mutation. Docking sites binding exclusively WT ERK2 conform to a distinct sequence motif from common binders. Through structural and biochemical studies, we identified molecular features dictating selective loss of binding to ERK2 mutants. These results establish rules allowing one to infer differentially interacting sequences by inspection. ERK2 substrates harboring these sequences are likely dispensable for tumorigenesis, and their decreased phosphorylation may underlie the transforming potential of cancer-associated ERK2 mutants.

## Results

### Defining the scope of docking interactions for ERK2 cancer mutants

To examine how cancer-associated CD site mutations affect the scope of ERK2 docking interactions, we adopted a Y2H strategy that we previously applied to define the D-site interactome of WT ERK2 (15). This system reports on interactions between ERK2 and a fragment of its substrate ELK1 that harbors a docking site essential for binding (Fig. 1*C*). The system thus evaluates the binding of potential sequences by substituting them for the native ELK1 docking site. We transformed Y2H strains with a bait plasmid expressing the Gal4 DNA-binding domain (DBD) fused to WT or CD mutant forms of rat ERK2 (R133K, D319N or E320K, equivalent respectively to human R135K, D321N and E322K), along with a prey plasmid expressing Gal4 activation domain (AD) fused to the ELK1 fragment harboring various docking site sequences. We then assayed these strains for growth on solid media under non-selective (+His) and selective conditions (−His) to detect interactions (Fig. 1*D*). Docking sequences from ELK1 itself (ELK1^WT^) or MAP2K2 (ELK1^MAP2K2-D^) appeared to interact with all ERK2 variants. Yeast growth rates associated with ELK1^WT^ were somewhat lower for each of the CD mutants in comparison to WT ERK2, suggesting reduced binding as has been reported previously for ERK2^D319N^ (31). Similarly, D319N and E320K mutation reduced, but did not eliminate, the interaction with ELK1^MAP2K2-D^. As controls, we observed no growth when the ELK1 D-site was mutated (ELK1^ΔD^) or substituted with a sequence selective for c-Jun N-terminal kinase (JNK) (ELK1^NFAT4-D^). Altogether, these results confirm the applicability of this system to define potential changes between WT and CD mutant ERK2 interactomes.

**Figure 2:**
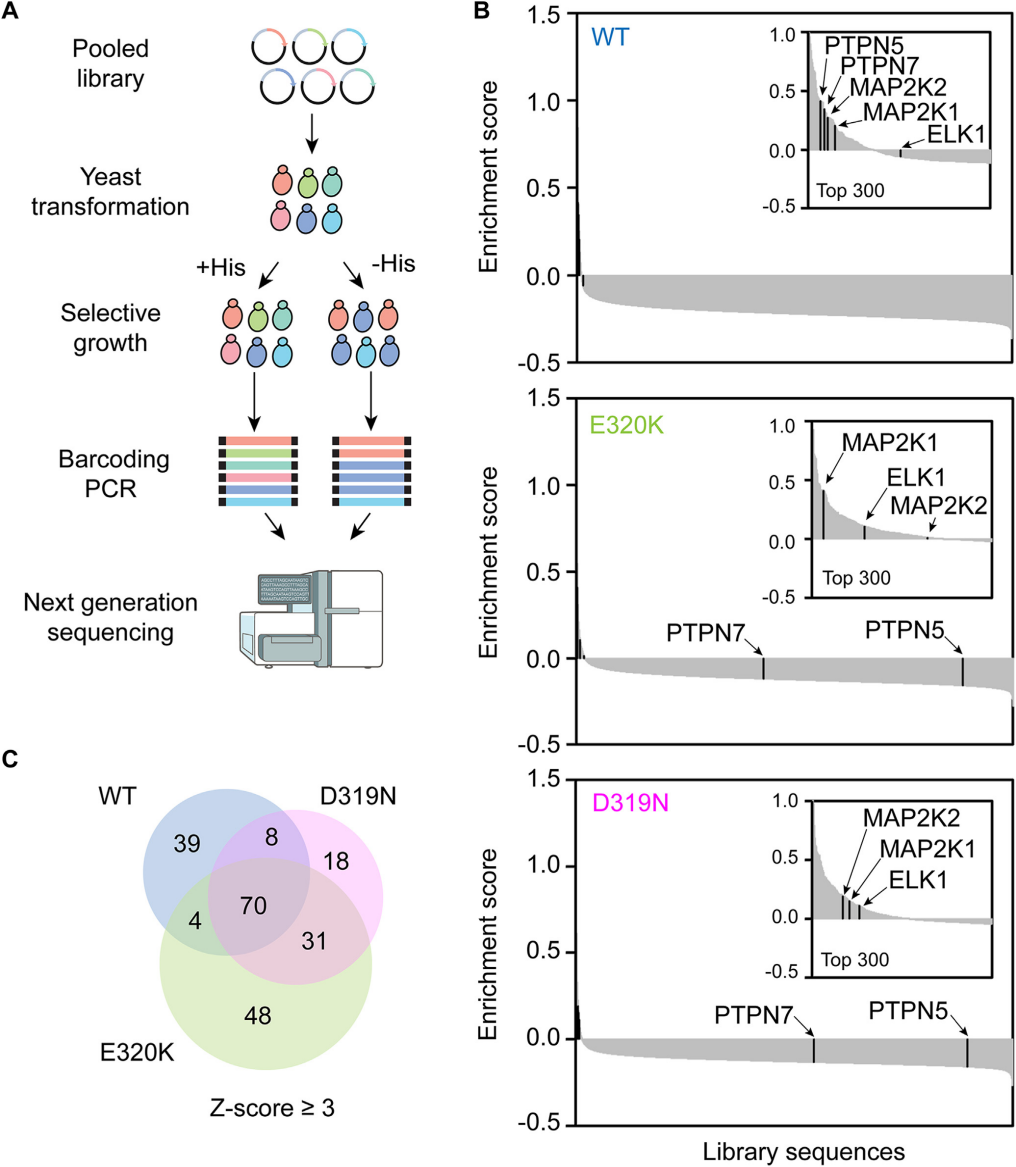
**Y2H Screening.***A*, Y2H screening workflow. The sequencer instrument icon is from NIAID NIH BIOART Source. *B*, waterfall plots depicting average ES from three independent screens for each D-site under selective conditions. Known ERK-interacting D-sites are labeled. Data for WT ERK2 was previously published (15) and shown for comparison. C, Venn diagram indicating overlap in hit sequences (ES Z-score > 3) for the WT and mutant ERK2 screens.

We previously used the Y2H system to screen WT ERK2 against a pooled library of 11,756 ELK1 variants incorporating potential docking sites from the human proteome (15). To identify interacting sequences either gained or lost upon ERK2 CD site mutation, we screened the same library against ERK2^D319N^ and ERK2^E320K^. Yeast expressing ERK2 mutant bait plasmids were transformed with the ELK1 library pool and subjected to competitive growth under selective (−His) and non-selective conditions (+His) in parallel. Cultures were sampled periodically over ∼7 population doublings. The DNA sequence encoding the variable docking site region was PCR amplified and analyzed by next-generation sequencing (Fig. 2*A*, Dataset S1). The change in sequence abundance over time was fitted to an exponential function to assign an enrichment score (ES) to each docking site, where a higher ES indicates a greater increase in sequence abundance as the population grows. A significant enrichment of docking site sequences was observed only under selective conditions (Figs. 2*B*, S1). For each mutant, we observed generally good reproducibility across three independently conducted screens albeit with some sequences enriched in only a single replicate (Fig. S2, *A* and *B*). Average ES scores for the E320K and D319N mutant screens were better correlated with each other (R = 0.83) than were average ES scores for either mutant compared to WT ERK2 (R = 0.67 for WT *versus* D319N, R = 0.63 for WT *versus* E320K) (Fig. S2*C*). This suggests that the ERK2 E320K and D319N mutants generally bind to a common pool of sequences and that a select number of sequences bind uniquely to WT.

Among the most enriched sequences selected by both CD mutants were the established D-sites from MAP2K1 and ELK1, known to retain interactions with ERK2^D319N^ and ERK2^E320K^ (Figs. 2*B*, S1). In contrast, D-site sequences from the phosphatases PTPN7 and PTPN5, which were enriched in screens with WT ERK2, were depleted in screens with both the D319N and E320K mutants (Fig. 2*B*). These results confirm that the screen recapitulates previously reported interaction patterns of CD mutants with activators, substrates, and regulators of ERK2 (26, 27).

To compare sequences selected by CD mutants with those selected by WT ERK2, we defined those sequences with an ES equal to or higher than three standard deviations from the mean (Z > 3) as hits. Using this cutoff, we obtained 121, 127, and 153 hits sequences from the WT, D319N, and E320K screens, respectively (Fig. 2*C*, Dataset S2). Categorizing sequences based on overlap between screens revealed most WT hits also interacted with at least one mutant, with only 32% being unique to WT ERK2. However, a similar fraction (31%) of sequences scoring as hits for both ERK2 mutants did not appear to interact with WT ERK2 (Fig. 2*C*). We interpret these results to suggest that CD site mutations may rewire docking specificity.

### Specificity determinants of ERK2 mutants D319N and E320K

**Figure 3:**
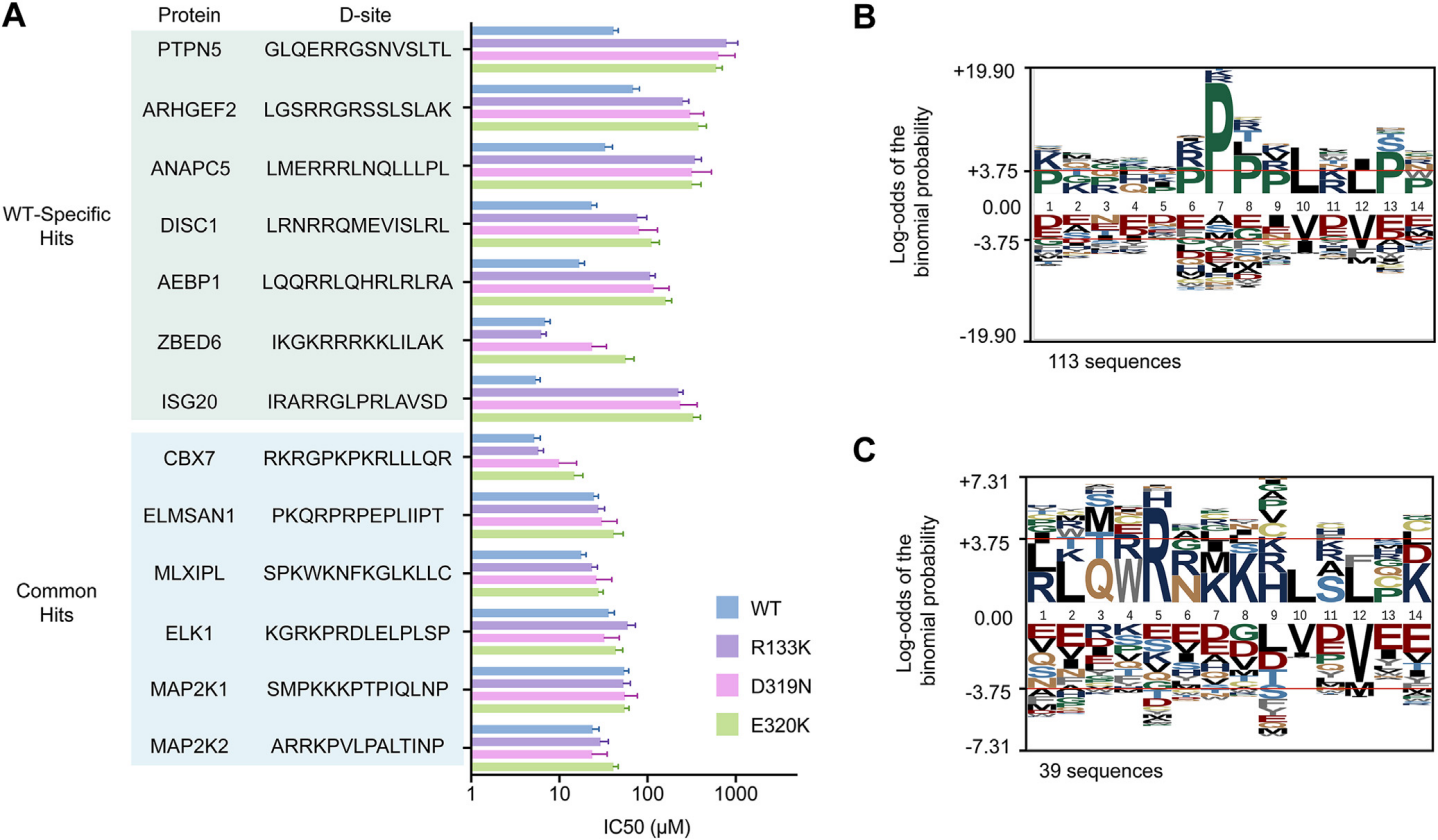
**Quantitative analysis of ERK-peptide interactions.***A*, bar graph showing average IC_50_ values for inhibition of ERK2 WT (*blue*), R133K (*purple*), D319N (*pink*), or E320K (*green*) with the indicated D-peptides. Error bars represent 95% CI from three independent experiments. One peptide (FAAP100) only interacted detectably with WT ERK2 and was excluded from the graph. Source data for all peptides are shown in Fig. S3 and calculated values are in Table S1. *B*, probability logo (pLogo) from multiple sequence alignment of common hits (Z > 3) across all screens. C, pLogo corresponding to hits unique to WT ERK2.

To verify binding patterns for WT and mutant ERK2 observed in our screens, we selected several hit D-site sequences for validation *in vitro* as chemically synthesized D-peptides. These included established mutant/WT interactors (ELK1, MAP2K2, MAP2K1), common top hits (ELMSAN1, MLXIPL, CBX7), seven lost interactors that bound only to WT ERK2 in all replicates, and five gained interactors binding only to one or both mutants. We determined relative binding affinities for D-peptides from their potency as competitive inhibitors of WT or mutant ERK2 activity on a D-site-dependent reporter substrate peptide (Table S1, Fig. 3*A*, S3). Most D-sites representing common hits bound all ERK variants with similar affinity (Fig. 3*A*). In contrast, all D-peptides encoding lost interactors had higher affinity for WT ERK2 over ERK2^D319N^ and ERK2^E320K^, with IC_50_ values ranging from 3 to 40-fold lower for the CD mutants (Fig. 3*A*, Table S1). We also examined D-peptide inhibition of ERK2^R133K^, another CD region cancer hotspot mutant that was not evaluated in our Y2H screens. With a single exception, peptides with decreased affinity for ERK2^D319N^ and ERK2^E320K^ likewise bound less tightly to ERK2^R133K^, suggesting a similar impact on docking specificity. Overall, these results verify that docking sites selectively scoring as hits in the WT ERK2 Y2H screen generally have reduced affinity for CD site mutants.

In addition to observing lost interactions, we also found 31 gained interactions, sequences scoring uniquely as hits for ERK2^D319N^ and ERK2^E320K^. However, when tested *in vitro* in competitive kinase assays, D-peptides corresponding to apparent gained interactors uniformly bound weakly to ERK2 and showed no preference for mutant forms over WT (Fig. S4*A*). We note that more than half (54%) of gained sequences would score as hits for WT ERK2 at a less stringent threshold (top 3% of sequences based on ES). It is thus possible that due to decreased competition from lost interactors, weakly binding sequences were not as substantially depleted from the population under selective growth conditions in the context of mutant ERK2. Finally, mutant-selective hits were not significantly enriched for any amino acid sequence features (Fig. S4*B*). These observations suggest that we did not reliably detect gained interactors in the Y2H screens, and accordingly, we focused our attention on WT-selective sequences.

To determine if WT-selective D-sites shared common sequence features, we aligned the corresponding sequences and conducted pLogo analysis to identify significantly selected residues at each position. Hit sequences common to any two screens displayed a significant overrepresentation of Pro in positions seven through 9 (Fig. 3*B*), consistent to our prior characterization of the full set of WT hits (15). These common sequences conform to previously reported ERK-binding motifs, including DCC (P-x-x-L-x-L), Far1 (P-x-P-L-x-L), or MEF2A (P-x-L-x-L) motifs (15). In contrast, we found the Pro-containing signatures to be absent from lost interactors, which instead displayed a significant overrepresentation of Arg at position five of the D-site library (Fig. 3*C*). Inspection of the sequences with the largest differential in ES values between WT mutant ERK2 revealed that 12 out of the top 20 conformed to the previously reported HEPTP motif (32), consisting of an I/L-x-x-R-R sequence near the N-terminus of the docking site (Table S2). For these sequences, the linker connecting the di-Arg and ϕ-x-ϕ motifs was variable, suggesting that they adopt distinct conformations when associated with the ERK2 docking groove. Notably, none of the 70 hits common to WT and either mutant conformed to this motif (Dataset S2).

### Crystal structure of ERK2 with ISG20 D-peptide

**Figure 4:**
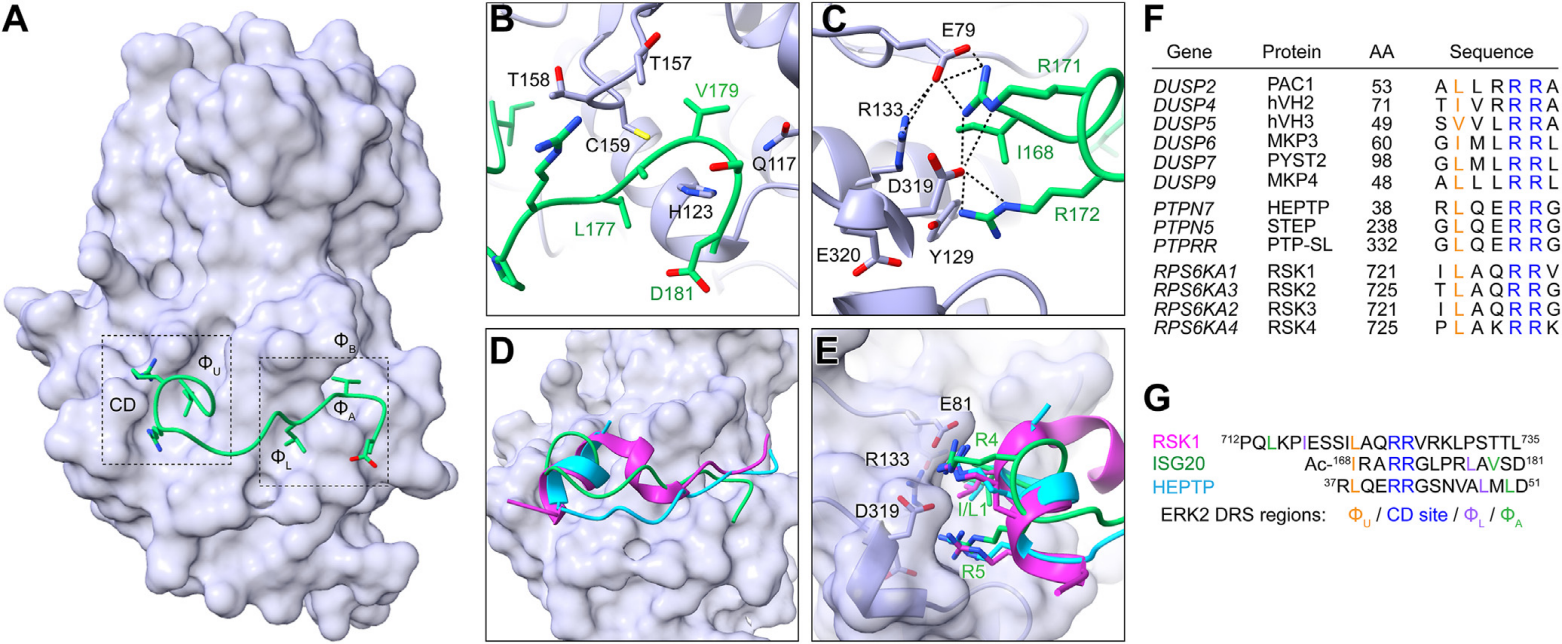
**Structural analysis of the complex between ERK2 and ISG20 D-peptide.***A*, co-crystal structure of ISG20-pep (*green*) and rat ERK2 (*lavender*). The common docking (CD) region and hydrophobic pockets (Φ_A_, Φ_B_, Φ_L_, and Φ_U_) are labeled. Peptide residue side chains engaging the ERK2 DRS are depicted as sticks. *B*, expanded view of interactions between the C-terminal portion of ISG20-pep and the ERK2 region including the β7-β8 loop (Thr157-Cys159) and the Φ_A_ and Φ_L_ pockets. *C*, zoomed in view of the extended interaction network involving the N-terminus of ISG20-pep and the CD region of ERK2. Hydrogen bonds as calculated using ChimeraX (54) are depicted as *dashed lines*. Refinement statistics are provided in Table 1. *D*, overlay of ERK2 complexes with peptides from ISG20 (*green*), RSK1 (*magenta*, PDB accession code: 3TEI) and HEPTP (*cyan*, PDB accession code: 2GPH). *E*, closeup of the CD region from panel D showing the I/L-x-x-R-R motif binding conformation of ISG20-pep, RSK1-pep, and HEPTP-pep. *F*, multiple sequence alignment shows conservation of I/L-x-x-R-R motif among human ERK-selective DUSPs, PTPs, and RSKs. G, Peptide sequences from overlaid structures in panels C and D are shown with the CD-interacting motif aligned, ERK2 DRS regions including the CD site and hydrophobic pockets (Φ) in contact with peptides are indicated at *bottom*.

To acquire structural insights into differential binding affinity between WT and CD mutant ERK2, we solved the co-crystal structure of WT ERK2 in complex with the ISG20 D-peptide (ISG20-pep, corresponding to residues 168 – 181 of human ISG20 with the sequence acetyl-IRARRGLPRLAVSD-amide). We chose the ISG20 sequence because it showed the largest differential interaction between WT and mutant ERK2 in biochemical assays (Fig. 3*A*). The co-crystal structure, containing a single copy per asymmetric unit, was determined to 1.9 Å resolution, with clear electron density corresponding to the D-peptide bound at the docking groove (Fig. S5*A*, Table 1). ISG20-pep binds the ERK2 docking groove in a largely extended conformation save for a single helical turn at the peptide N-terminus, including the HEPTP motif I-x-x-R-R sequence (Fig. 4*A*). At the C-terminus, ISG20-pep residues Leu177′ and Val179′ occupy the hydrophobic pockets Φ_L_ and Φ_A_ in ERK2, mostly forming hydrophobic contacts with residues in αE and the β7-β8 loop (Figs. 4*B*, S5*C*). Similar to other reported MAPK D-peptide complexes, backbone atoms of ISG20-pep Ala178′ and Val197′ engage in a series of polar contacts with Thr167 backbone and the sidechains of Gln117 and His123 in ERK2 (14, 16, 32, 33). Interestingly, unlike all other MAPK D-peptide complexes solved to date, the Φ_B_ pocket is unoccupied, likely due to the absence of a hydrophobic residue at the corresponding position in ISG20-pep (Asp181′).

**Table 1:** ERK2-ISG20-pep crystal complex data collection and refinement statistics

Parameter	ERK-ISG20-pep PDB ID: 7UGB
Data Collection	
Wavelength (Å)	0.97918
Resolution range (Å)^a^	43.97–1.90 (1.97–1.90)
Space group	*P*2_1_2_1_2_1_
Cell dimensions	
a, b, c (Å)	45.89, 66.68, 116.97
α, β, γ (°)	90.0, 90.0, 90.0
Unique reflections	29,145 (2866)
Multiplicity^a^	23.7 (15.4)
Completeness (%)^a^	100 (100)
Mean *I/σ (I)*^a^	29.9 (2.0)
Wilson *B* factor (Å^2^)	34.7
*R*_pim_ (%)^a^	2.6 (30.8)
CC ½^a^	0.998 (0.828)
CC^a^	1.000 (0.952)
Refinement Statistics	
Resolution Range (Å)	43.97–1.90
Reflections used in refinement	29,020 (2686)
Reflections used for *R*_free_	1451 (138)
*R*_work_ (%)^a^	17.9 (23.7)
*R*_free_ (%)^a^	22.8 (27.1)
No. of non-hydrogen atoms	
Protein-ERK2	2736
Ligand-ANP-PNP	31
Peptide-ISG20-pep	113
Solvent-H2O	146
Protein Residues	333
RMSD	
Bond lengths (Å)	0.006
Bond angles (°)	0.804
Ramachandran plot (%)	
Favored, allowed, outliers	97.3, 2.7, 0.0
Rotamer outliers	0.00
MolProbity clashscore	0.17
Average B factor (Å^2^)	
Protein-ERK2	46.7
Ligand-ANP-PNP	49.9
Peptide-ISG20	64.7
Solvent-H2O	45.3

a Parentheses indicate highest resolution shell.

### Impact of cancer hotspot mutations on binding specificity

At the ISG20-pep N-terminus, the Ile168′ sidechain occupies the upper hydrophobic pocket (Φ_U_) of ERK2 (comprising Glu81, Tyr126, Gln130, Arg133). Spatially adjacent to Ile168′, Arg171′ and Arg172′ make salt bridges with Glu79 and Asp319 of ERK2. This explains why cancer hotspot mutations at Glu79 and Asp319 disrupt binding to HEPTP motif sequences (Fig. 4*D*). Similarly, Arg133, though not directly contacting the bound peptide, coordinates Glu79, likely stabilizing peptide binding (Fig. 4*C*). Lastly, cancer hotspot residue Glu320, as previously indicated by the crystal structure of ERK2 E320K, appears to stabilize the ERK2 L16 loop conformation (24) and consequently orient Asn319 for contact to Arg171′ and Arg172′ in the ISG20-pep. Collectively, these observations suggest that disruption of direct and indirect interactions through cancer-associated ERK2 mutations causes selective loss of interactions with D-sites harboring an I/L-x-x-R-R motif.

To test this hypothesis, we evaluated a set of synthetic ISG20 D-peptides containing single amino acid substitutions or insertions for binding to WT and mutant ERK2 using the competitive kinase assay described above (Table 2). We observed that amino acid substitutions in positions 1, 4, and 5 (ISG20^I1N^, ISG20^R4A^, and ISG20^R5A^) within the I-x-x-R-R motif caused ∼40-fold reduction in affinity towards WT ERK2, consistent with prior mutagenesis experiments showing that the analogous residues promote high affinity binding of full length RSK1, PTPN5, and MKP3 to ERK2 (34, 35, 36, 37). Substituted peptides bound equally to WT and mutant forms of ERK2, indicating that CD site mutation causes no further decrease in affinity for sequences lacking the I-x-x-R-R motif. We observed a similar effect when we either decreased (ISG20^I1R/R2I^) or increased (ISG20^G2+^) the spacing between Ile1 and the Arg4-Arg5 sequence by one residue, suggesting that positioning of these key residues is essential for optimal and differential binding to WT ERK2. Extending the distance between I-x-x-R-R sequence and the canonical D-site hydrophobic motif (ϕ-x-ϕ) by adding a single Gly in position 7 (ISG20^G7+^) caused a 15-fold reduction in affinity to WT ERK2. Finally, we noted a Gly residue was overrepresented at the position immediately following the I/L-x-x-R-R in sequences binding preferentially to WT ERK2 (Table S2). Consistent with this observation, we found that ISG20^G6Q^ displayed an 8-fold lower affinity towards ERK2 than ISG20^WT^ yet showed increased binding to all three CD site mutants (Table 2). These results establish a conserved structural motif associated with differential binding to WT ERK2.

**Table 2:** IC_50_ values with for ISG20-pep variants as determined in competitive kinase assays

ISG20 peptide	Sequence	ERK2 (μM)							
		WT		R133 K		D319 N		E322 K	
		IC_50_	95% CI	IC_50_	95% CI	IC_50_	95% CI	IC_50_	95% CI
WT	IRARRGLPRLAVSD	5.4	4.8–6.2	220	198–253	230	187–290	330	270–399
I1N	**N**RARRGLPRLAVSD	190	168–204	180	154–211	230	181–287	260	224–291
R4A	IRA**A**RGLPRLAVSD	250	215–285	380	310–470	320	263–395	300	249–368
R5A	IRAR**A**GLPRLAVSD	250	215–304	480	398–585	440	361–551	400	320–508
I1R/R2I	**RI**ARRGLPRLAVSD	200	185–215	220	193–259	310	268–368	280	243–322
G2+	I**G**RARRGLPRLAVSD	120	107–125	150	130–167	150	125–185	170	143–202
G7+	IRARRG**G**LPRLAVSD	75	71–80	170	152–198	150	122–180	210	181–242
G6Q	IRARR**Q**LPRLAVSD	41	38–43	99	85–115	93	77–113	150	129–167

The mean and 95% confidence interval (CI) are shown for three independent replicates.

The importance of this motif and its unique specificity to WT ERK2 are also highlighted in previously reported co-crystal structures of ERK2 and D-peptides from HEPTP (PTPN7) and RSK1, as well as structural and biochemical analyses of MAPK interactions with the folded kinase interacting domains of DUSP-type MKPs. As for ISG20, the docking sites of RSK1, HEPTP, and ERK-binding DUSPs contain a conserved I/L-x-x-R-R motif (Fig. 4, *E*–*G*). When overlayed at the ERK2 DRS, the short peptide binders (ISG20, RSK1, and HEPTP) adopt unique overall binding modes (Figs. 4, *D-G* and S6). However, the common I/L-x-x-R-R motif adopts an almost identical helical conformation to establish the same H-bonding network with charged residues Glu79, Arg133, and Asp319 in the CD site. A similar structural motif is observed in the co-crystal structure of the MAPK p38α in complex with the N-terminal MAPK-binding globular domain from MKP5 (DUSP10) (38), which we have recapitulated in an AlphaFold 3 model of ERK2 bound to MKP3 (Fig. S6, *C* and *D*). In these structural models, part of the binding interface involves a helical I/L-x-x-R-R sequence in precisely the same orientation as in the ISG20 complex, with remaining contacts with the DRS found at sites discontinuous in primary sequence. These observations suggests that this helical binding mode defines the specificity of the I/L-x-x-R-R motif for WT ERK2.

### ERK2 CD mutants show reduced phosphorylation of GEF-H1

**Figure 5:**
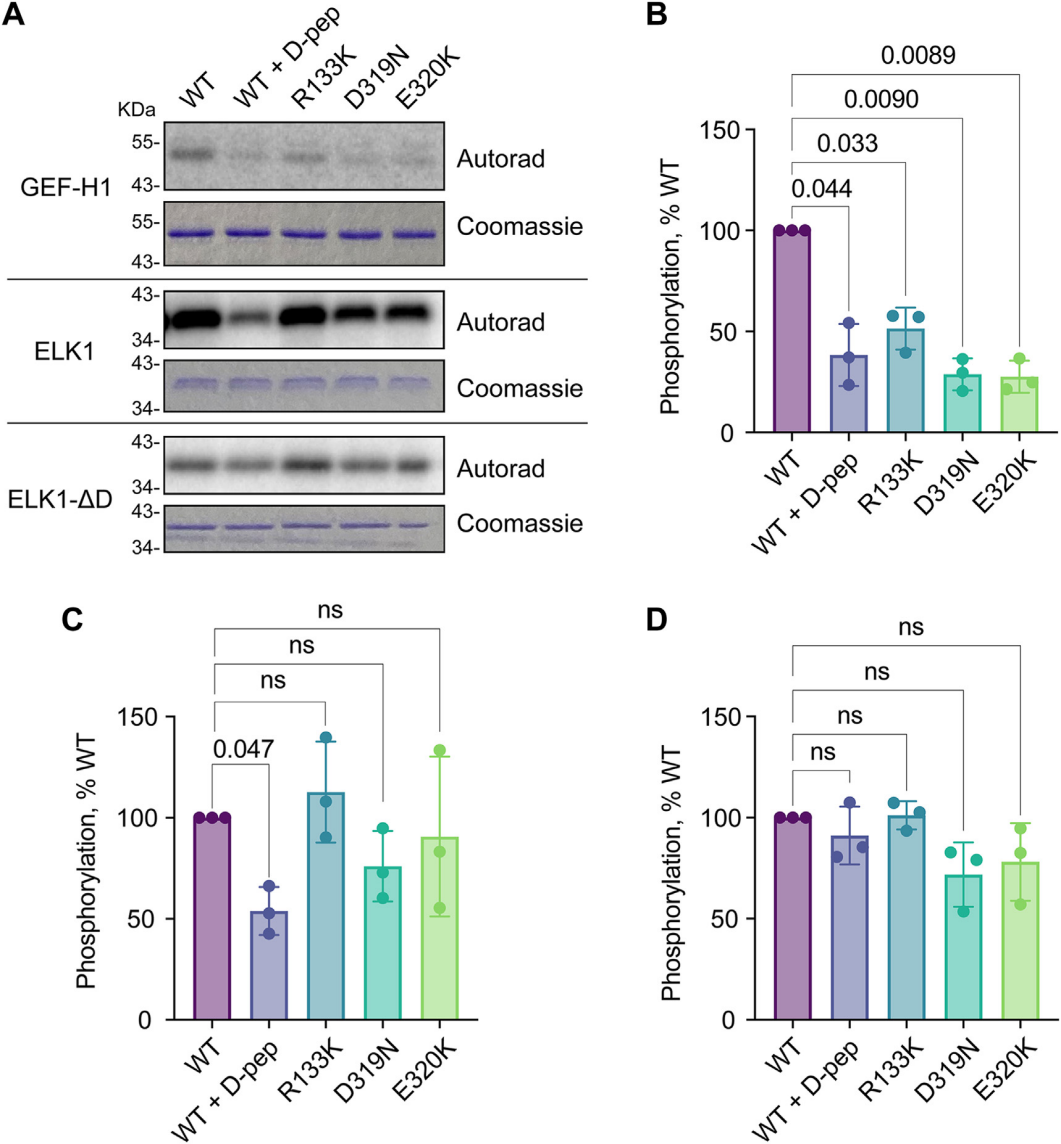
**ERK2 phosphorylation of GEF-H1 *in vitro*.***A*, autoradiography and Coomassie-stain images from radiolabel kinase assays of ERK2 (WT or mutant) mediated phosphorylation of GEF-H1, WT ELK1, and previously reported ELK1 docking impaired mutant (ΔD) (44). D-pep indicates an excess of the KMT2C D-peptide was included in the reaction. Each substrate was assayed separately. A single representative of three separate experiments is shown. *B–D*, quantified phosphorylation levels from the radiolabel kinase assays in panel A. The *p* values were calculated by ordinary one-way ANOVA with Geisser-Greenhouse correction and Dunnet's multiple comparisons test using PRISM. Datapoints are from three independent replicates. Bars show mean ± SD.

Based on our structural and mutational analysis, we hypothesized that ERK2 CD site mutants should not bind or efficiently phosphorylate validated ERK2 substrates containing an I/L-x-x-R-R D-site motif. We previously reported that while ISG20 interacts physically with ERK2 in cells, it is not an ERK2 substrate *in vitro* (15). However, other WT-selective hits containing I/L-x-x-R-R motifs included the D-site from the established ERK substrate GEF-H1 (encoded by the *ARHGEF2* gene), which *in vitro* bound WT ERK2 approximately 4-fold more tightly than the three ERK2 CD mutants (Fig. 3*A* and Table S1). We previously reported that disruption of the GEF-H1 docking motif reduced its extent of co-immunoprecipitation with ERK2 (15). Accordingly, we investigated whether cancer-associated ERK2 mutants were impaired in phosphorylating GEF-H1. In radiolabel kinase assays, the phosphorylation of GEF-H1 by all three CD site mutants was significantly lower than that of WT ERK2 (Fig. 5, *A* and *B*). The level of phosphorylation by CD site mutants was similar to that of WT ERK2 when assayed in the presence of excess competing D-peptide to block docking interactions. For comparison, we examined phosphorylation of the ERK2 substrate fragment of ELK1 (residues 307–428), whose D-site lacks the I/L-x-x-R-R motif, as well as its corresponding D-site mutant (ΔD) form (Fig. 5, *A*, *C* and *D*). As expected, the competing D-peptide partly inhibited WT ERK2 phosphorylation of WT ELK1 but did not significantly affect phosphorylation of ELK1-ΔD. In contrast to GEF-H1, phosphorylation of neither WT ELK1 nor ELK1-ΔD was significantly affected by any of the CD site mutations. We interpret these results to confirm that ERK2 CD mutants can disrupt a functional interaction with a full-length protein substrate harboring the WT-selective I/L-x-x-R-R motif.

## Discussion

Here, we used a Y2H screening approach to define the docking interactome of the ERK2 cancer-associated CD site mutants D319N and E320K. We identified an I/L-x-x-R-R motif as diagnostic for docking sites for which these mutations disrupt binding. In the co-crystal structure of WT ERK2 in complex with the ISG20 D-peptide, this motif adopted a helical conformation, with the rest of the peptide binding in an extended conformation in the DRS. Interestingly, the binding mode of this peptide was distinct from that seen in other MAPK D-peptide complexes (Fig. 4, *B* and *D*) (13, 14, 16, 32). In particular, the Φ_B_ pocket was unoccupied likely owing to the absence of a hydrophobic residue at the corresponding position in the peptide. Consistent with this observation, a report from Bardwell and co-workers indicated only a modest effect of ϕ_B_ residue mutation on binding of some D-sites to p38α or ERK2. Furthermore, in our own positional scanning analysis of JNK1 binding to the MKK7 D-site, we found a hydrophobic residue at the ϕ_B_ position to be less critical than at the ϕ_L_ and ϕ_A_ positions, suggesting that this phenomenon may be a general feature of MAPK D-site interactions (17, 18). The limited requirement for a hydrophobic residue at this position has implications for discovering MAPK docking sites by *in silico* scanning of protein sequences.

A comparison of our ERK2-ISG20 peptide complex with prior structures reveals that ISG20 binds to the docking groove with a distinct overall conformation but has intriguing similarities in the region containing the I/L-x-x-R-R and engaging the CD pocket. For example, the HEPTP D-peptide binds ERK2 in a clamp-like conformation. Similar to ISG20-pep, the helical N-terminus engages the CD pocket, but in contrast the C terminal region binds in a more extended mode (Figs. 4*D*, Fig. S6*A*) (32). On the other hand, the RSK1 D-peptide binds ERK2 in reverse orientation, with its helical C-terminus interacting with the CD pocket (Figs. 4, *E* and *F* and S6*B*). In contrast to ISG20 and HEPTP, the N-terminal segment of the RSK1 D-peptide bends and adopts a helical conformation to place hydrophobic residues into the Φ_B_ and Φ_L_ pockets (16). Finally, unlike the short linear motif interactions adopted by most D-sites, the MKPs bind to the MAPK DRS region *via* a large interaction surface involving their N-terminal kinase interacting domain (38). This folded domain includes a helix harboring an I/L-x-x-R-R sequence bound to the CD site. Strikingly, when the ISG20, HEPTP, RSK1, and MKP docking sites are overlayed in the ERK2 docking pocket, it is evident that despite having distinct overall conformations, their I/L-x-x-R-R sequences make almost identical direct and indirect contacts with cancer hotspot residues (Figs. 4, *D* and *E*, S6, *C* and *D*). Thus, regardless of the overall interaction mode, a helical I/L-x-x-R-R sequence motif appears to be a common feature of docking interactors requiring an intact CD region. While this motif has been challenging to identify by scanning the human proteome *in silico*, partly because it can be found in two different orientations, we and others have identified this sequence in essential ERK2 interacting proteins (14, 32). The importance of this sequence motif is highlighted by its conservation across species (Fig. S7). Altogether our structural studies provide a rationale for how these cancer-associated mutants may rewire signaling through the proteins harboring D-sites with a I/L-X-X-R-R motif requiring strong stabilizing interactions with the CD pocket in ERK.

In our screen, the majority of WT ERK2 binders (70%) also scored as hits for ERK2 mutants. Most of these common hit sequences conformed to previously defined docking motif classes, including the DCC (P-x-x-L-x-L) and MEF2 (P/L-x-L-x-I/L-P) types (14). We note that, at least for some known binders corresponding to these motifs, the presence of multiple basic residues at the N-terminal end of the motif is critical for MAPK binding (39). However, in co-crystal structures with ERK2 or other MAPKs, the N-terminal basic residues often cannot be modeled, suggesting that they lack stable structure even in crystals. In these cases, specific fuzzy interactions driven by bulk electrostatics may be more important than the formation of stable ion pairs, potentially explaining why mutation of only a single acidic residue in the CD region does not fully compromise binding. In some ERK2 complexes, such as those with MAP2K2 and ELK1 peptides, only a subset of acidic residues at the CD site appear to contact the bound peptide (33, 40). In these cases, mutation of individual residues could induce conformational rearrangement, forming alternate ion pairs that maintain binding affinity. Indeed, increased disorder in the L16 loop caused by the E322K mutation (24) may increase accessibility of nearby acidic residues, such as Asp318, allowing them to engage basic residues in docking sites.

The unique interactomes of ERK2 CD site mutations raise questions with respect to their roles as cancer drivers. Perhaps unexpectedly, ERK2 hotspot mutations are rarely found in tumor types with high-frequency mutations in other core MAPK pathway components (melanoma) or upstream RAS proteins (colorectal, lung, and pancreas cancer). We speculate that the capacity of ERK signaling to function in oncogenesis depends on substrates or other interactors specific to WT ERK2, such as RSK or GEF-H1. This idea is supported by recent observations that D321N mutation renders ERK2 incapable of promoting growth upon KRAS inhibition in pancreatic cancer cells (30). The prevailing model for how ERK2 CD mutations drive cancer is through disrupting its interaction with MAPK phosphatases, leading to its persistent activation. However, CD mutations could also affect signaling dynamics or other systems-level properties. For example, substrate competition is established in model systems to influence ERK signaling (41). As RSKs are relatively abundant proteins, disrupting their interaction may allow ERK better access to other docking partners, such as MEK. Similarly, the oncogene adipocyte enhancer-binding protein 1 (AEBP1), which we identified as a differential binder, was reported to compete with MAPK phosphatases for binding to ERK and likely restricts its access to other interaction partners as well (42). While the scope of interactions lost by the two mutants evaluated here were similar to each other, we did observe some sequences binding preferentially to either ERK2^D319N^ or ERK2^E320K^, which may relate to reported functional divergence between mutants in a *Drosophila* model (24). Further studies should inform whether different cancer hotspot mutants contribute to tumors in divergent ways.

This work and previously reported studies emphasize the importance of being mindful when using CD site mutants as tools to study ERK function. Because the equivalent mutation was identified as a GOF allele in *Drosophila* and it is known to be hyperphosphorylated in cells, ERK^D321N^ has been used as a constitutively active allele to increase ERK pathway signaling. Conversely, the same mutant has often been used to establish or rule out a role for DRS interactions in an ERK-dependent process. Our work systematically shows that ERK2 CD mutations can disrupt interactions with specific proteins, including negative regulators, and maintain interactions with many essential regulators. Accordingly, D319N and other CD mutants should be regarded as both partial GOF and partial loss-of-function alleles. True intrinsically active ERK mutants have been reported that are unlikely to interfere with substrate interactions and are thus better suited for GOF studies (43). To our knowledge, mutants that generally block D-site interactions have not been reported but will likely require neutralizing multiple charged residues in the docking region.

Overall, our work builds upon the general knowledge of how docking interactions can drive specificity and cellular signaling. Because these conserved docking sequences mainly occur within intrinsically disordered protein regions, they can be difficult to discover, limiting our understanding of how point mutations can rewire ERK2 signaling to promote cancer. This work serves as a resource for identifying WT and mutant ERK docking interactions, which can be used to accelerate docking sequence discovery *in silico* and to identify unknown network connections that may drive disease progression in tumors harboring ERK2 mutants.

## Experimental procedures

### Plasmids

Bacterial expression constructs for GST-ELK1^WT^ (mouse, residues 307–428), GST-ELK1^ΔD^ (L320A/E321A/L322A mutant), and His_6_-tagged GEF-H1 (human, residues 29–959) were reported previously (44). ELK1^307–428^ Y2H prey plasmids (pGADGH-ELK1, pGADGH-ELK1^ΔD^, pGADGH-ELK1^MEK2D^, pGADGH-ELK^NFAT4D^, and ELK1 D-site library pool) and the Y2H bait plasmid (and pGBT9-ERK2) were described previously (15). The D-site library replaces the native docking sequence of ELK1 with a set of 14-mer sequences found within disordered regions of the human proteome conforming to the consensus sequence [R/K]-x_0-2_-[R/K]-x_3-5_-[ILV]-x-[FILMV] (15). The Y2H bait plasmid and bacterial expression plasmid (pET28a-ERK2^WT^) were used as templates to generate ERK2^D319N^, ERK2^E320K^, and ERK2^R133K^ by QuikChange mutagenesis. All primers are listed in Table S3.

### Y2H growth assay

The yeast host strain pJ694-A (45) was sequentially transformed with the indicated bait (pGBT9-ERK2) and prey (pGADGH-ELK1) plasmids. Cultures from single colonies were grown in SD-Leu-Trp media in a shaking incubator overnight at 30 °C, diluted to OD_600_ of 0.1, and cultured to OD_600_ of 1.0. Five-fold serial dilutions (starting at OD_600_ = 0.5) were spotted onto agar plates with non-selective (SD-Leu-Trp) or selective (SD-Leu-Trp-His) media supplemented with 50 μM 3-amino-1,2,4-triazole (3-AT) and incubated 4 days at 30 °C.

### Y2H library screen

Yeast harboring pGBT9-ERK2^E320K^ or pGBT9-ERK2^D319N^ were transformed with the pGADGH-ELK1 D-site library plasmid pool. Transformants (10–40 colonies per library component) were suspended in SD-Leu-Trp media, diluted to an OD_600_ of 0.1, and cultured in a shaking incubator at 30 °C to an OD_600_ of 1.0. The resulting culture was used to initiate a competitive selection screen. Cell growth, sample collection and data analysis were performed exactly as described previously for WT ERK2 (15). The frequency (F) of each D-site sequence at a given time point (t) was calculated by dividing the number of reads by the total read count for all sequences. For each sequence, a plot of ln(F_t_/F_0_) as a function of the number of yeast population doublings was fit to a line, and the enrichment score (ES) for that sequence was defined as the slope of this line. Illumina sequencing reads, calculated ES, and Z scores for each screen are provided in Dataset S1.

### Protein expression and purification

Recombinant His_6_-GEF-H1, GST-ELK1^WT^, and GST-ELK^ΔD^ were expressed in *E. coli* and purified as previously described (15, 44). Briefly, proteins were expressed in Rosetta DE3 pLysS *E. coli* (Invitrogen) using IPTG induction (0.4 mM) for 12 to 18 h at 16 °C. Bacterial cells were harvested, resuspended in lysis buffer (20 mM Tris, [pH 7.5], 140 mM NaCl, 3 mM β-mercaptoethanol, 10 mM imidazole, 2 μg/μl pepstatin A, 10 μg/μl leupeptin), and lysed *via* sonication. The lysates were clarified by centrifugation at 3000*g*, then incubated with Talon immobilized metal affinity resin (Takara #635502) for 2 h at 4 °C. After incubation, the beads were washed twice with wash buffer (20 mM Tris [pH 7.5], 150 mM NaCl, 0.01% Igepal CA-630, 10 mM imidazole), and proteins were eluted with the same buffer supplemented with 250 mM imidazole. Finally, proteins were dialyzed overnight in dialysis buffer (20 mM HEPES [pH 7.4], 150 mM NaCl, 10% glycerol, 1 mM DTT) and stored at −80 °C. Active phosphorylated His_6_-tagged ERK2 variants (WT, D319N, E322K, and R133K) were produced by co-expression with GST-MEK1ΔN3 in BL21(DE3) *E. coli* by induction with 0.4 mM IPTG overnight at 30 °C in LB plus kanamycin (33 μg/ml). Bacterial pellets from 1L cultures were resuspended in 25 ml of lysis buffer (as above but including 200 μg/ml lysozyme, 0.5% Igepal CA-630, 1 mM PMSF, 0.03 U/μl DNAse I and 13 mM MgCl_2_), and lysed by sonication. Lysates were clarified and proteins isolated on Talon resin, dialyzed, and stored as described above.

To generate ERK2 for X-ray crystallography, eight x 1L cultures of BL21(DE3) *E. coli* cells co-expressing His_6_-ERK2 and λ phosphatase (from pACYC-LIC) were grown as described above, and cells were pelleted, and resuspended in 25 ml lysis buffer per 1L culture (50 mM sodium phosphate [pH 8.0], 300 mM NaCl, 0.2 mg/ml lysozyme, 0.5% Triton X100, 0.03 U/μl DNAse I, 13 mM MgCl_2_, 1 mM PMSF, 10 μg/ml leupeptin, 2 μg/ml pepstatin A) and lysed by sonication. Lysates were clarified by centrifugation for 30 min at 3000*g* and incubated with Talon beads for 1 h at 4 °C. Beads were washed five times with 10-bed volumes of wash buffer (50 mM sodium phosphate [pH 8.0], 300 mM NaCl, 40 mM imidazole) and protein eluted in 6 ml of elution buffer (50 mM sodium phosphate [pH 8.0], 300 mM NaCl, 300 mM imidazole [pH 8.0]). The eluate was filtered and dialyzed overnight into buffer A (10 mM Tris HCl [pH 8.2], 0.5 mM TCEP). Dialyzed protein was applied to a Mono Q 5/50 Gl column (GE Healthcare) equilibrated to buffer A and eluted with a gradient of 0 to 25% buffer B (10 mM Tris HCl [pH 8.2], 0.5 mM TCEP, 1 M NaCl) over 40 column volumes. The peak eluting first was collected and applied to a size exclusion column (Superdex 200 10/300 Gl, GE Healthcare) in buffer C (25 mM Tris HCl [pH 7.5], 1 mM TCEP, 100 mM NaCl, 1 mM EDTA). Fractions were pooled and concentrated with a centrifugal filter (Amicon Ultra, 3000 MWCO) to 6 to 8 mg/ml, flash frozen, and stored at −80 °C.

### X-ray crystallography

ISG20-pep used for co-crystallography, corresponding to residues 168 to 181 of human ISG20 (acetyl-IRARRGLPRLAVSD-amide), was synthesized and HPLC purified at the Tufts University Core Facility and stored as a 50 mg/ml aqueous stock solution. Unphosphorylated His_6_-ERK2 (7.6 mg/ml), ISG20-pep (2x molar excess), 2 mM ANP-PNP and 2 mM MgCl_2_ were incubated on ice for 10 min. Crystallization was performed by hanging-drop vapor diffusion. The conditions for crystallization were found using the PEG II suite (position E4) (Qiagen) containing 10% PEG 4000, 0.1 M HEPES [pH 7.5] and 5% isopropanol as the reservoir solution. The best crystals were achieved by mixing the reservoir and protein-peptide mix in 1:1 volume ratio at room temperature. Individual crystals were harvested, preserved in a reservoir solution supplemented with 20% glycerol, and flash-frozen in liquid nitrogen.

Diffraction data were collected at the Advanced Photon Source, beamline 24-ID-E (Argonne National Laboratory, IL) and indexed, integrated, and scaled using HKL2000 (46) in space group *P*2_1_2_1_2_1_ with unit cell dimensions a = 45.9 Å, b = 66.7 Å, c = 117.0 Å, α = β = γ = 90°, to a 1.90 Å resolution and one molecule per asymmetric unit. The protein structure was determined by molecular replacement using Phaser (47, 48) with PDB 4FMQ (14) as a search model. The model was iteratively refined with TLS, stereochemistry and ADP weight optimization in phenix.refine (48). After refinement in Phenix, the resulting sigma 2*F*o-*F*c at one sigma and *F*o-*F*c difference map at 2.5 sigma were sufficient to clearly define atoms in the ligand (ANP-PNP) and side chains in the docking peptide (ISG20-pep). Based on these maps, manual model building was performed in Coot (49) and validated by phenix.refine (48) and MolProbity (50). The final refinement statistics are *R*_free_ 17.9% and *R*_work_ 22.8% (Table 1).

### Molecular interaction model predictions

The structure of full length ERK2 in complex with MKP3/DUSP6 was modeled with the AlphaFold 3 web server using default parameters (51).

### Peptide kinase assay

D-peptides used in competitive kinase assays were synthesized commercially (GenScript) with an added N-terminal Tyr-Ala sequence to facilitate quantification by A_280_ and stored as 50 mM solutions in DMSO. Competitive peptide kinase assays were performed with a D-site dependent MAPK reporter substrate peptide (AssayQuant Technologies Inc, #AQT0376) as we reported previously (15). Briefly, 5 μM of reporter peptide was mixed with various concentrations of D-peptides in reaction buffer (54 mM HEPES [pH 7.4], 1.2 mM DTT, 0.01% Brij-35, 1% glycerol, 0.2 mg/ml BSA, and 10 mM MgCl_2_). Reactions were initiated by adding 10 nM of recombinant mouse ERK2 (WT, D319N, E322K or R133K) and 10 μM ATP. Phosphorylation of the reporter peptide was measured by fluorescence (λ_ex_ 360 nm, λ_em_ 485 nm) in 90 s intervals over 21 min in a Spectra M5 plate reader. Rates were calculated from the linear portion of the reaction progress curve, and data were normalized to maximum (samples lacking competitor peptide) and minimum (control reactions lacking kinase) values. Normalized rates were plotted against D-peptide concentration and fitted into a sigmoidal dose-response curve to determine the IC_50_ values using GraphPad Prism (Version 9.3.0).

### Radiolabel kinase assays

Radiolabel kinase assays were performed as described previously by incubating 0.5 μM MAPK substrate (His_6_-GEF-H1, GST-ELK1^WT^ or GST-ELK1^ΔD^), 10 nM His_6_-ERK2 (WT, R133K, D319N or E320K) and 10 μM inhibitor peptide KMT2C where indicated (biotin-RKRSKPKLKLKIIN-amide, Tufts University Core Facility) in reaction buffer (20 mM HEPES, 10 mM MgCl_2_, 1 mM DTT, 1% DMSO, 10 μM cold ATP, and 0.038 μCi/μl [γ-^32^P] ATP) at 30 °C for 2 min. Reactions were quenched by the addition of 4x SDS-PAGE loading buffer and heated to 95 °C for 5 min before SDS-PAGE fractionation. Gels were stained with Coomassie blue, destained, dried, and exposed to a phosphor screen. Radiolabel incorporation was measured with Molecular Imager FX phosphorimager (Bio-Rad), and ^32^P signal density (CNT/mm^2^) was quantified using QuantityOne Software (Bio-Rad version 11.0.5). All signals were normalized to the vehicle control condition. Raw quantified data are provided in Dataset S3.

## Data availability

All screening datasets generated in this study are provided in the supplementary material as Dataset S1. The dataset for ERK2 WT Y2H has been previously reported (15). Crystal structure coordinates and structure factors have been deposited in the Protein Data Bank under accession code 7UGB and X-ray diffraction images are available online at SBGrid Data Bank (52), under Data set 883 (https://doi.org/10.15785/SBGRID/883). Next generation sequencing data from Y2H screens has been deposited in the Gene Expression Omnibus (GEO) repository under accession codes: GSE286340 (ERK2 E320K and D319N Mutants) and GSE286341 (WT ERK2).

## Supporting information

This article contains supporting information (51, 55, 56).

## Conflict of interest

The authors declare that they have no conflicts of interest with the contents of this article.
