# Supplementary material for: Cancer hotspot mutations rewire ERK2 specificity by selective exclusion of docking interactions
Source: J Biol Chem. 2025 Feb 25;301(4):108348. doi: 10.1016/j.jbc.2025.108348 (PMC11982978; doi:10.1016/j.jbc.2025.108348)
Supplement: Supporting information [file mmc4.pdf]

SUPPORTING INFORMATION

Cancer hotspot mutations rewire ERK2 specificity by selective exclusion of docking interactions

Jaylissa Torres Robles, Amy L. Stiegler, Titus J. Boggon, Benjamin E. Turk

Contents:

Figure S1. Lack of D-site enrichment in Y2H screens under control non-selective conditions.

Figure S2. Y2H screen reproducibility and comparisons between screens.

Figure S3. D-peptide kinase inhibition assay dose-response curves.

Figure S4. D-peptide kinase inhibition assay dose-response curves for mutant-specific hits.

Figure S5. ISG20-pep interactions with ERK2 CD pocket.

Figure S6. Interaction modes of I/L-x-xR-R motifs with ERK2.

Figure S7. I/L-x-x-R-R motif sequence conservation.

Table S1. IC50 values from competitive kinase binding assay.

Table S2. WT-selective sequences ranked by differential ES.

Table S3. Oligonucleotides used in this study.

Inventory of supporting datasets

Supporting references

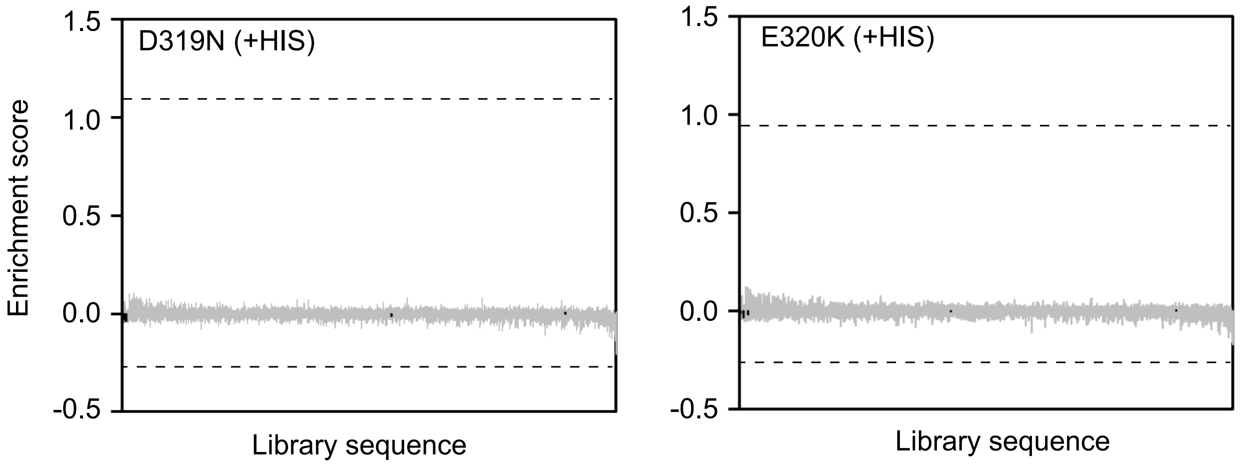

Figure S1. Lack of D-site enrichment in Y2H screens under control non-selective conditions. Waterfall plots showing average enrichment scores (ES) for each library member in D319N and E320K ERK2 mutant screen under non-selective (+His) control growth conditions. Dotted lines indicate top and bottom ES values from the corresponding screens conducted under selective (-His) conditions shown in Figure 1B. D-sites are sorted in the same order as in Fig. 2B.

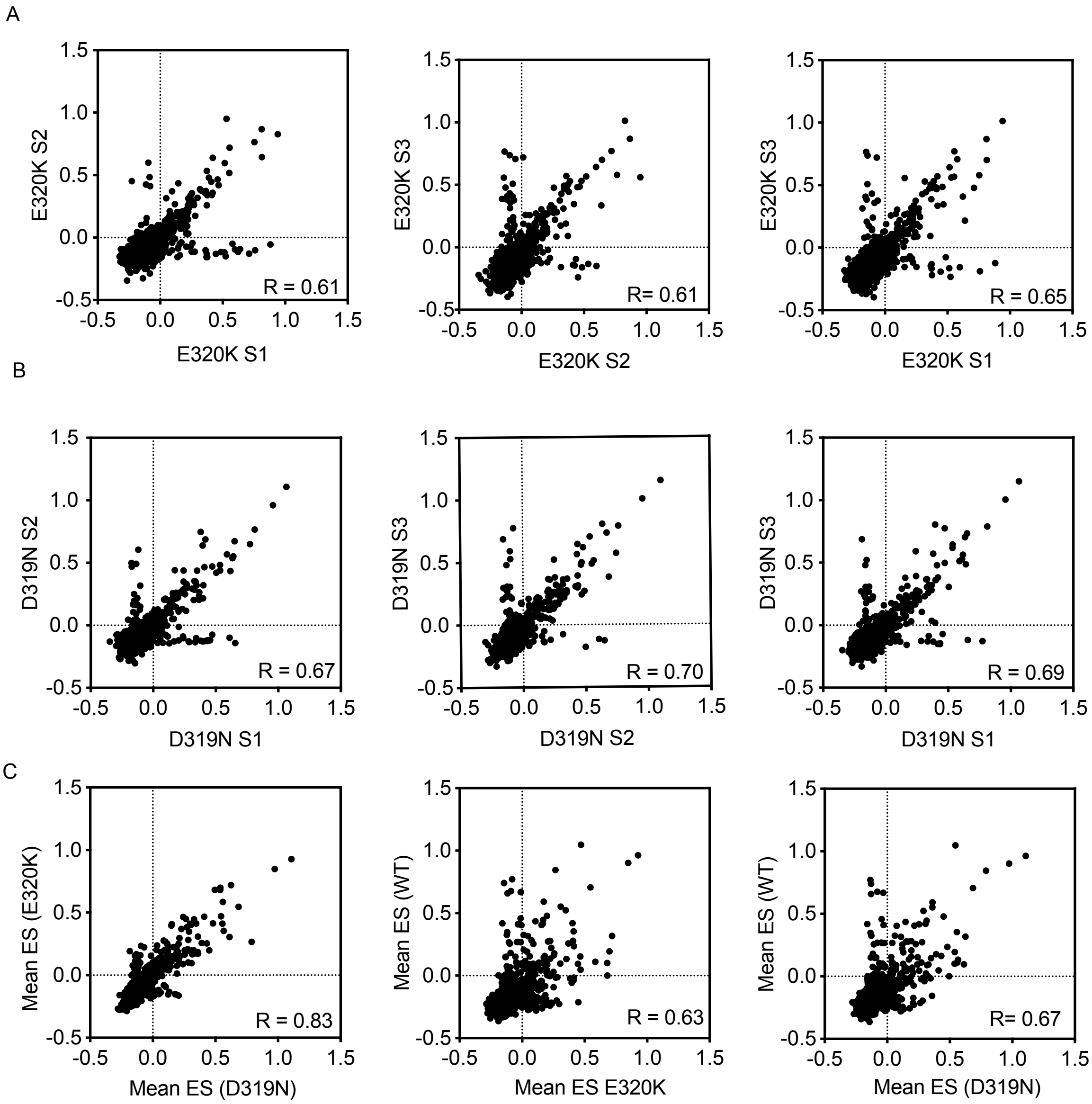

Figure S2. Y2H screen reproducibility and comparisons between screens. A-B, ES correlations for each pairwise combination of three independent replicate screens with ERK2 E320K (A) and D319N (B). Each datapoint is a single sequence in the Y2H library. C, pairwise correlations of the mean ES score for each library component between the three screens. Pearson R is shown for all correlations.

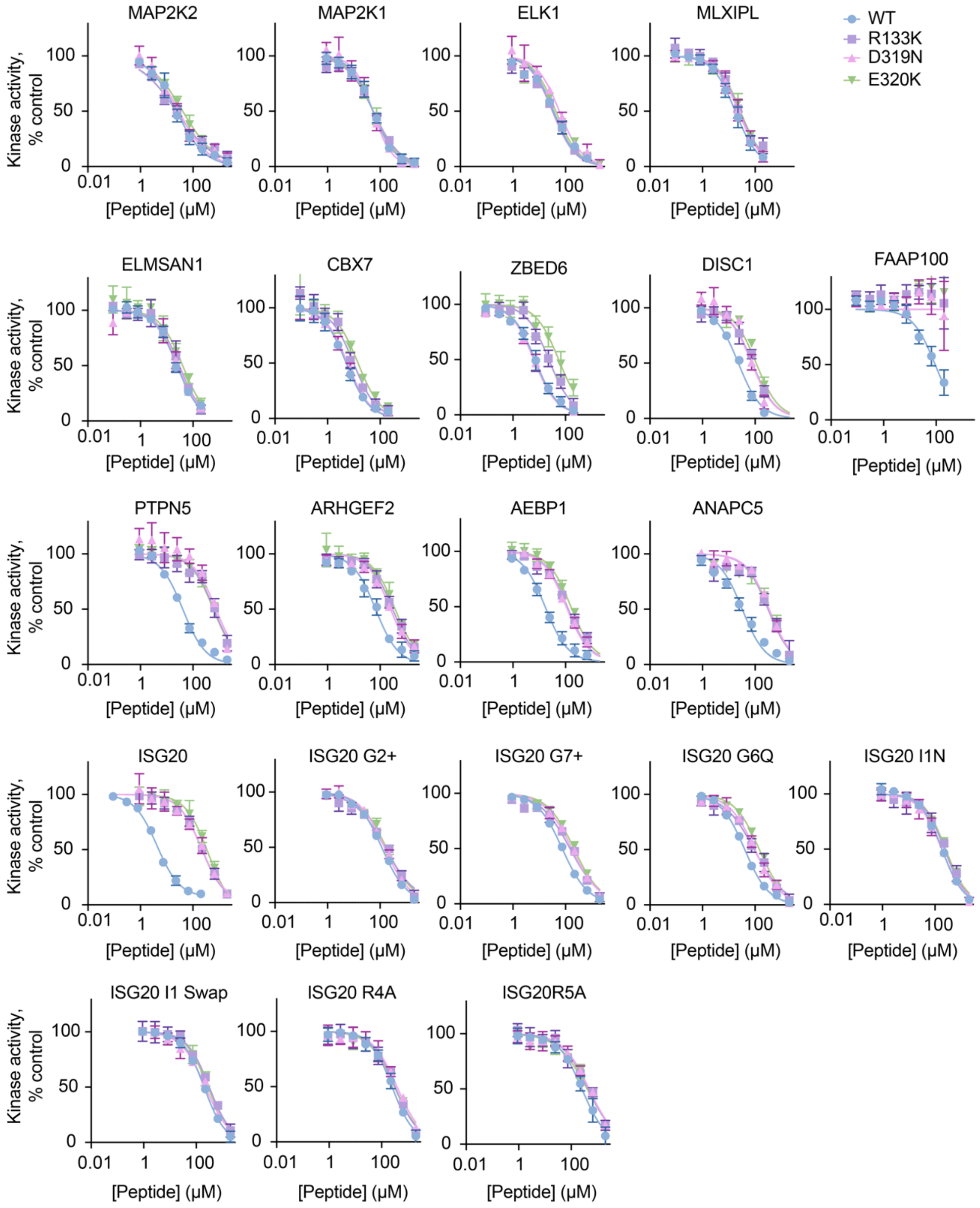

Figure S3. D-peptide kinase inhibition assay dose-response curves. Dose-response curves for indicated D-peptides for competitive inhibition of ERK WT(blue, circle), R133K (purple, square), D319N (pink, upward triangle), or E320K (green, downward triangle). Data points represent the mean from three replicate experiments. Error bars represent SD. Calculated IC_{50} values are tabulated in Table 1 and Table S1 and were used for the bar graphs in Fig. 3A and Fig. 4D.

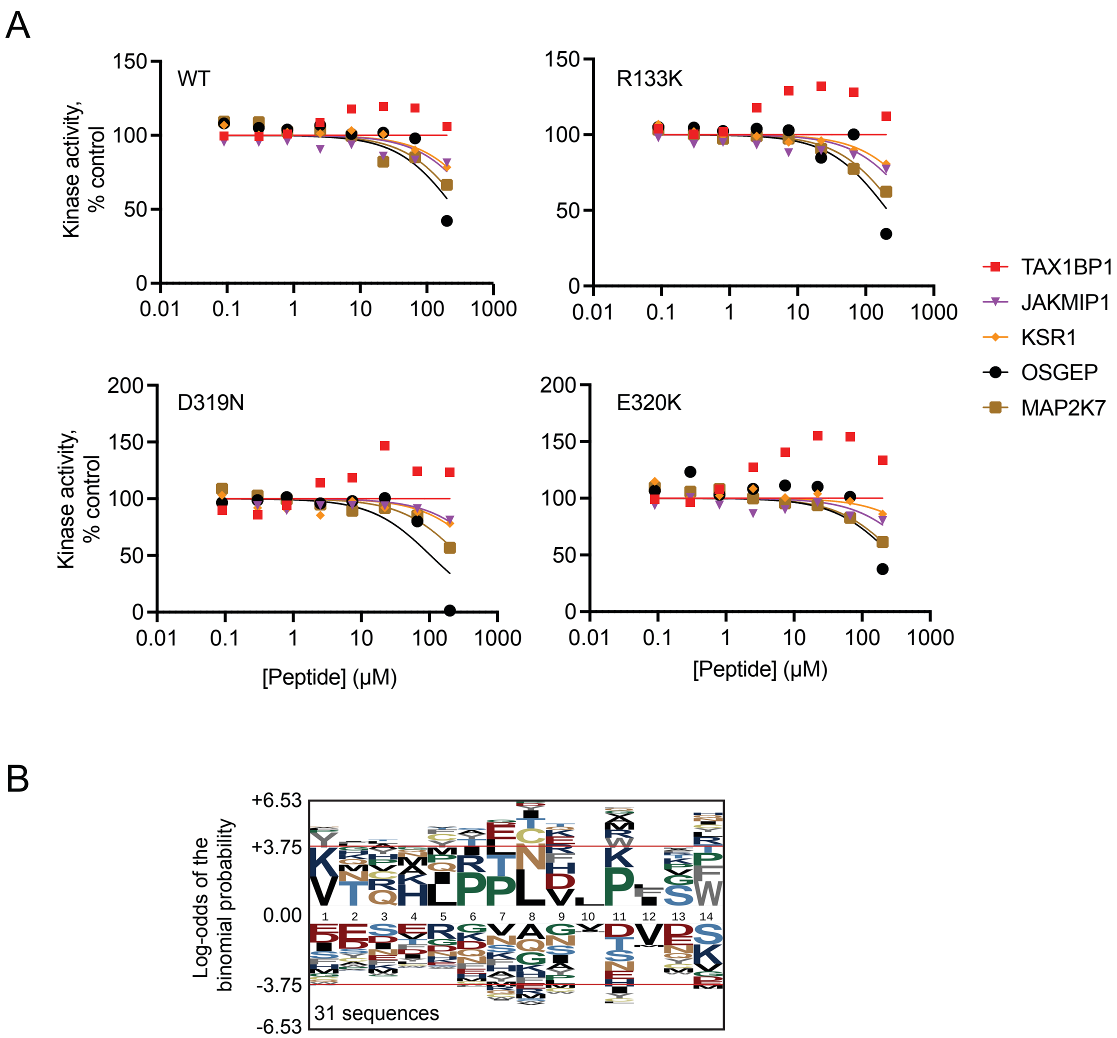

Figure S4. D-peptide kinase inhibition assay dose-response curves for mutant-specific hits. A, Dose-response curves for ERK2 WT or mutant kinase activity inhibition by D-peptides representing gained interactors in the Y2H screens with mutant ERK2. B, Probability logo generated from multiple sequence alignment of the 31 hit sequences common to D319N and E320K ERK2 mutants only.

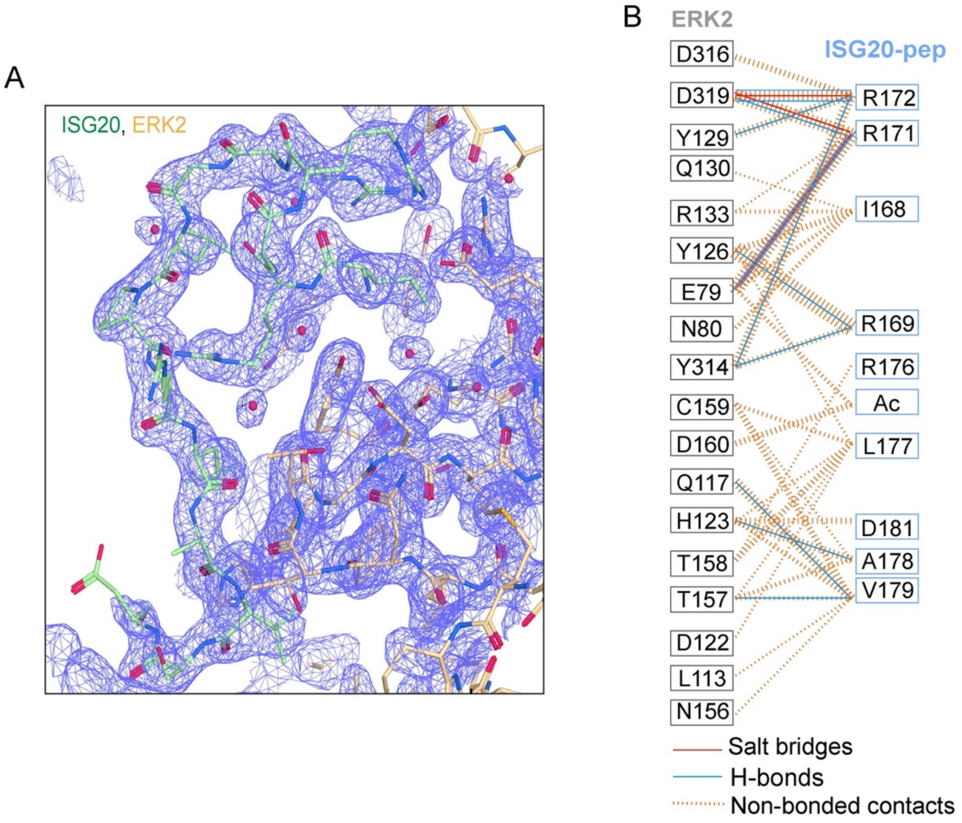

Figure S5. ISG20-pep interactions with ERK2 CD pocket. A, Sigma A weighted 2Fo-Fc map depicting the electron density contoured at 1.03 sigma in the interaction region between ISG20-pep (green) and the ERK2 docking pocket (beige), visualized using moorhen.org. B, Map summarizing ERK2-ISG20-pep intermolecular interactions (generated with PDBsum).

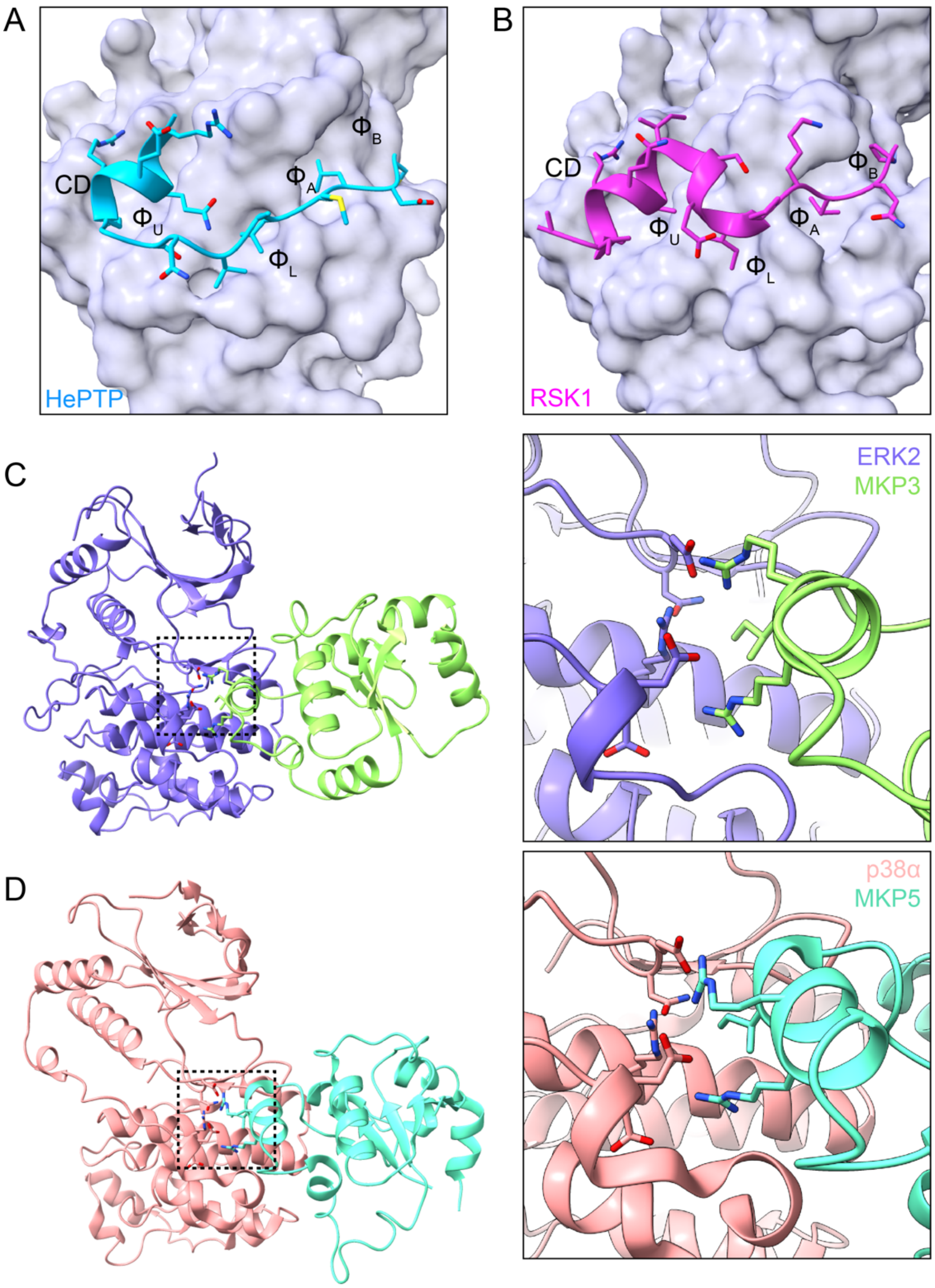

Figure S6. Interaction modes of I/L-x-xR-R motifs with ERK2. A, Structure of RSK1-pep in complex with ERK2 (PDB entry 3TEI). B, ERK2 in complex with HEPTP-pep (2GPH). C, AlphaFold3 (1) model of ERK2 bound to the N-terminal kinase binding domain of MKP3/DUSP6. D, X-ray crystal structure of p38α bound to the N-terminal kinase binding domain of MKP5/DUSP10 (3TG1).

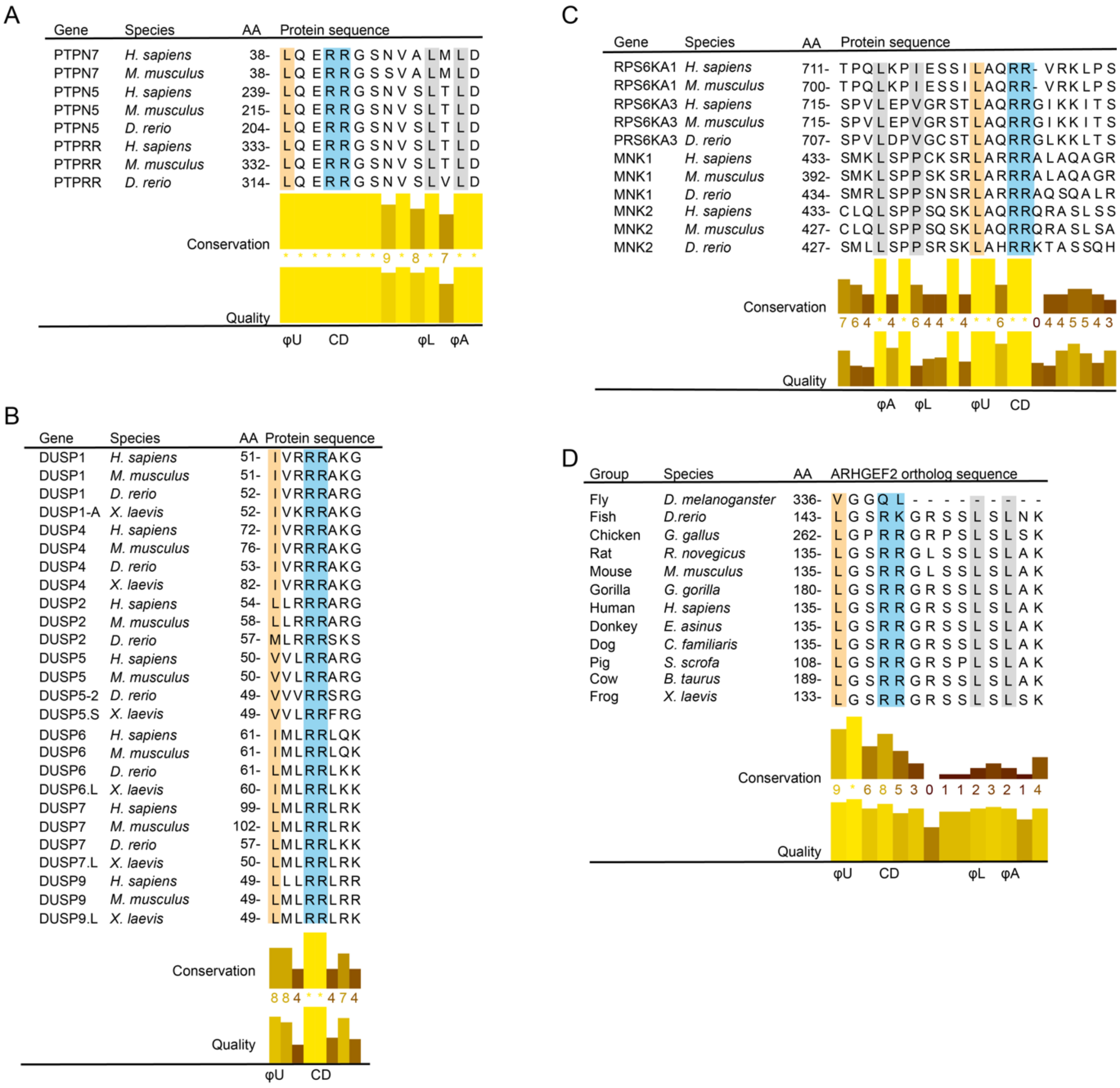

Figure S7. I/L-x-x-R-R motif sequence conservation. Clustal Omega sequence alignments (2,3) of I/L-x-x-R-R regions of ERK binding partners from the indicated species. A, ERK specific protein tyrosine phosphatases. B, Dual specificity phosphatases. C, RSK family ERK substrates. D, ARHGEF2/GEF-H1 orthologs.

Table S1. IC50 values from competitive kinase binding assay.

Peptide	Sequence	ERK2							
		WT		R133K		D319N		E322K	
		IC_{50} (μM)	95% CI	IC_{50} (μM)	95% CI	IC_{50} (μM)	95% CI	IC_{50} (μM)	95% CI
AEBP1	LQQRRLQHRLRLRA	16	14.4-19.3	110	92.1-123	120	94.9-142	160	135-189
ANAPC5	LMERRRLNQLLLPL	32	26.2-40.2	340	285-411	310	239-408	310	239-407
ARHGEF2	LGSRRGRSSLSLAK	67	55.7-81.3	250	211-295.	300	253-358	370	299-467
CBX7	RKRGPKPKRLLLQR	5.1	4.35-6.08	5.7	4.99-6.62	9.8	7.74-12.3	14	11.3-18.6
DISC1	LRNRRQMEVISLRL	23	20.1-26.5	74	55.7-99.1	79	61.1-101	110	87.7-137
ELK1	KGRKPRDLELPLSP	35	30.4-42.1	58	46.6-73.0	32	26.3-38.6	43	35.1-52.5
ELMSAN1	PKQRPRPEPLIIPT	24	21.8-27.7	27	23.0-32.7	30	24.8-36.4	40	30.8-53.3
FAAP100	LCARRGLYCLSLDH	92	72.4-118	>200	-	>200	-	>200	-
MAP2K1	SMPKKKPTPIQLNP	54	47.4-61.1	52	42.9-63.9	55	46.8-63.9	55	48.3-61.6
MAP2K2	ARRKPVLPALTINP	23	19.6-28.2	29	22.9-36.0	23	19.2-28.2	41	35.7-46.5
MLXIPL	SPKWKNFKGLKLLC	18	15.4-20.1	23	19.8-27.0	26	21.2-31.7	28	24.4-31.4
PTPN5	GLQERRGSNVSLTL	42	35.9-46.4	760	538-1070	600	509-783	600	505-704
ZBED6	IKGKRRRKKLILAK	6.8	5.96-7.88	6.2	5.45-7.09	23	19.3-27.9	56	42.9-70.4

Table S2. WT-selective sequences ranked by differential ES. Mutant ES is the combined average ES from the D319N and E320K screens.

Gene name	Start residue	Sequence	HePTP motif	Arg-Arg-Gly	WT ES - mutant ES
ANAPC5	57	LMERRRLNQLLLPL	+	-	0.878
AEBP1	1021	LQQRRLQHRLRLRA	+	-	0.878
GLB1L3	14	CLSWKRMAGIFFLP	-	-	0.781
ISG20	169	IRARRGLPRLAVSD	+	+	0.761
PTPN5	242	GLQERRGSNVSLTL	+	+	0.577
LIG3	52	PLFLRRKPVLSFQG	+	-	0.487
PTPN7	41	RLQERRGSNVALML	+	+	0.474
FAAP100	82	LCARRGLYCLSLDH	+	+	0.450
ANKRD12	1111	RLRNRNCLELKIKD	-	-	0.443
ZNF675	189	TRHERNYTKVNFCK	-	-	0.359
TNRC18	1996	LWTRRRSERIFLHD	+	-	0.332
ASPM	1508	LYKRRKESILTIQK	+	-	0.329
HAS3	68	RRMRRAGQALKLPS	-	-	0.310
POLR3C	361	GSRCARIFRLVLQK	-	-	0.285
KLC2	62	ILLRRSLEAIELGL	+	-	0.282
ABCA7	285	RLLWRRLKPLILGK	+	-	0.266
ZNF493	630	HLSRHKIIHIGIHT	-	-	0.221
DISC1	343	LRNRRQMEVISLRL	+	-	0.195
TACC1	229	RSKLRKPKPVPLRK	-	-	0.162
BAZ1A	275	RRRGRPPKRIHISQ	-	-	0.150

Table S3. Oligonucleotides used in this study.

Sequence	Description
CTATTTTCTTTATCAGATCCTGAAAGGATTAAAGTATATACATTC	QuikChange rat ERK2 R133K forward
GAATGTATATACTTTAATCCTTTCAGGATCTGATAAAGAAAATAG	QuikChange rat ERK2 R133K reverse
GTATTATGACCCAAGTGATAAGCCCATTGCTGAAGC	QuikChange rat ERK2 E320K forward
GCTTCAGCAATGGGCTTATCACTTGGGTCATAATAC	QuikChange rat ERK2 E320K reverse
CAGTATTATGACCCAAGTAATGAGCCCATTGCTGAAG	QuikChange rat ERK2 D319N forward
CTTCAGCAATGGGCTCATTACTTGGGTCATAATACTG	QuikChange rat ERK2 D319N reverse
AATGATACGGCGACCACCGAGATCTACACTCTTTCCCTACACGACGCTCT TCCGATCTNNNNNNNNCGATGGGTCGCGGATCTATGTCTCAG	Illumina Hi Seq_F1
AATGATACGGCGACCACCGAGATCTACACTCTTTCCCTACACGACGCTCT TCCGATCTNNNNNNNNGCCAAGGTCGCGGATCTATGTCTCAG	Illumina Hi Seq_F2
CAAGCAGAAGACGGCATACGAGATATTATAGTGACTGGAGTTCAGACGTG TGCTCTTCCGATCGAAGGAGAGCTTGGCTGGAC	Illumina Hi Seq_R1 i7index R744
CAAGCAGAAGACGGCATACGAGATGAATGAGTGACTGGAGTTCAGACGT GTGCTCTTCCGATCGAAGGAGAGCTTGGCTGGAC	Illumina Hi Seq_R2 i7index R745
CAAGCAGAAGACGGCATACGAGATTCGGGAGTGACTGGAGTTCAGACGT GTGCTCTTCCGATCGAAGGAGAGCTTGGCTGGAC	Illumina Hi Seq_R3 i7index R746
CAAGCAGAAGACGGCATACGAGATCTTCGAGTGACTGGAGTTCAGACGT GTGCTCTTCCGATCGAAGGAGAGCTTGGCTGGAC	Illumina Hi Seq_R4 i7index R747
CAAGCAGAAGACGGCATACGAGATTGCCGAGTGACTGGAGTTCAGACGT GTGCTCTTCCGATCGAAGGAGAGCTTGGCTGGAC	Illumina Hi Seq_R5 i7index R748

Supporting datasets

Dataset S1. Illumina sequencing reads and calculated ES for Y2H screens.

Dataset S2. Hit sequences from Y2H screens.

Dataset S3. Autoradiography quantification

Supporting References

1. Abramson, J., Adler, J., Dunger, J., Evans, R., Green, T., Pritzel, A., Ronneberger, O., Willmore, L., Ballard, A. J., Bambrick, J., Bodenstein, S. W., Evans, D. A., Hung, C. C., O'Neill, M., Reiman, D., Tunyasuvunakool, K., Wu, Z., Zemgulyte, A., Arvaniti, E., Beattie, C., Bertolli, O., Bridgland, A., Cherepanov, A., Congreve, M., Cowen-Rivers, A. I., Cowie, A., Figurnov, M., Fuchs, F. B., Gladman, H., Jain, R., Khan, Y. A., Low, C. M. R., Perlin, K., Potapenko, A., Savy, P., Singh, S., Stecula, A., Thillaisundaram, A., Tong, C., Yakneen, S., Zhong, E. D., Zielinski, M., Zidek, A., Bapst, V., Kohli, P., Jaderberg, M., Hassabis, D., and Jumper, J. M. (2024) Accurate structure prediction of biomolecular interactions with AlphaFold 3. *Nature* **630**, 493-500
2. Goujon, M., McWilliam, H., Li, W., Valentin, F., Squizzato, S., Paern, J., and Lopez, R. (2010) A new bioinformatics analysis tools framework at EMBL-EBI. *Nucleic Acids Res.* **38**, W695-699
3. Troshin, P. V., Procter, J. B., and Barton, G. J. (2011) Java bioinformatics analysis web services for multiple sequence alignment--JABAWS:MSA. *Bioinformatics* **27**, 2001-2002
